# Golden Anniversary Celebration

**Published:** 1895-12

**Authors:** 


### GOLDEN ANNIVERSARY CELEBRATION.

The attention of the Dental Profession in Philadelphia is called to the notice under this head in "Current News.'

The past fifty years has witnessed greater changes for the advancement of dentistry than any previous period, and it therefore seems proper that it should be celebrated by the joint efforts of the dental societies in that city. It is anticipated that the occasion will be one of interesting reminiscences of an epoch replete with activity.

